# IL-2 Receptor Antagonist (Basiliximab) Is Associated with Rapid Fibrosis Progression in Patients with Recurrent Hepatitis C after Liver Transplantation Using Serial Biopsy Specimens

**Published:** 2010-02-01

**Authors:** I. A. Hanouneh, N. N. Zein, R. Lopez, L. Yerian, J. Fung, B. Eghtesad

**Affiliations:** 1 *Department of Internal Medicine, *; 2 *Department of Gastroenterology and Hepatology, *; 3 *Department of Quantitative Health Sciences,*; 4 *Department of Anatomic Pathology, *; 5 *Department of General Surgery, Transplant Center, The Cleveland Clinic, Cleveland, Ohio, USA*

**Keywords:** Transplantation, liver, interleukin receptor antagonist, hepatitis C virus, liver fibrosis, cirrhosis

## Abstract

Background: Recurrence of hepatitis C virus (HCV) infection following orthotopic liver transplantation (OLT) is universal. There is paucity of data on the safety and efficacy of interleukin (IL)-2 receptor antagonist (IL-2RA) when added to the standard immunosuppression regimen in OLT recipients with recurrent HCV infection.

Objectives: To evaluate the efficacy of IL-2RA (Basiliximab) in preventing acute cellular rejection (ACR) in patients with recurrent HCV infection after OLT and to assess the impact of IL-2RA in promoting fibrosis progression in post-OLT recurrent HCV infection.

Methods: Using an electronic pathology database, we identified all OLT/HCV patients with at least 2 post-OLT liver biopsies (1998–2006). Standard immunosuppression consisted of steroids and calcineurin inhibitor with and without mycophenolate mofetil. All patients who were transplanted after May 2004 received IL-2RA induction therapy. The Ludwig-Batts system was used to stage all biopsies (593 biopsies from 124 patients). The first biopsy that showed post-OLT fibrosis or the last follow-up biopsy was used for time-to-progression analysis. Univariate and multivariate Cox proportional hazards regression analyses were performed to identify factors associated with the progression of fibrosis.

Results: ACR was significantly (p<0.001) lower in patients who received IL-2RA (20 of 70, 29%) compared to those who did not (33 of 54, 61%). The median (25%ile, 75%ile) follow-up was 12.1 (6.1, 23.9) months during which 61% of patients had progression of fibrosis. Univariate analysis revealed that a higher HCV RNA load at 4 months post-OLT (p=0.002), cytomegalovirus (CMV) infection (p<0.001), use of steroid therapy for ACR (p=0.043), and use of IL-2RA (p<0.001) were associated with higher hazards for the progression of fibrosis. Viral load at 4 months post-OLT was significantly (p=0.025) higher in patients who had IL-2RA therapy (median [25%ile, 75%ile]: 2.9 [1.0, 5.0] ×10^6 ^ vs. 1.4 [1.0, 2.3] ×10^6^). In multivariate analysis, patients who received IL-2RA therapy were 3.1 (95% CI: 1.8–5.3) times more likely to develop fibrosis than those who did not treated with IL-2RA. Steroid therapy for ACR remained significantly (Hazard Ratio=2.9, p=0.002) associated with the progression of fibrosis.

Conclusion: IL-2RA (Basiliximab) decreases the rate of ACR. However, it may be associated with more rapid histological progression of the disease in post-OLT recurrent HCV.

## INTRODUCTION

Chronic infection with hepatitis C virus (HCV) continues to be the most common indication for orthotopic liver transplantation (OLT) in the United States and Western Europe. Recurrence of HCV infection after liver transplantation is a universal event with serious consequences including cirrhosis and liver failure. It is estimated that 20% to 40% of HCV-positive liver transplant recipients develop cirrhosis by five years post-OLT [1, 2], and once cirrhosis occurs, the one-year risk of hepatic decompensation reaches 40% [1-3]. The identification of modifiable factors that promote more severe recurrence of HCV infection is therefore of paramount importance for any strategy aims at improving the outcomes.

Several donor, recipient, and viral factors influence the severity of HCV recurrence following liver transplantation. Advanced donor age [4], post-transplant diabetes mellitus [5], and high pre- and post-transplant viral load are associated with more rapid progression of fibrosis and allograft failure [6, 7]. Cytomegalovirus (CMV) infection has also been associated with increased severity of HCV recurrence [8]. Immunosuppressant agents, particularly, corticosteroids and lymphocyte depleting agents such as OKT3 and ATG are associated with higher levels of HCV viremia, and more severe histological recurrence [9-12]. In contrast, the choice of calcineurin inhibitor [12-14] and the use of mycophenolate mofetil (MMF) [15-17] have not been clearly shown to affect the severity of recurrence in HCV-infected liver recipients.

Interleukin (IL)-2 receptor antagonist (IL-2RA) has been shown to reduce the frequency of acute cellular rejection (ACR) in renal transplant recipients without increased incidence of adverse events [18]. However, conflicting reports obscure the value of IL-2RA when added to the standard immunosuppression regimen in liver transplant recipients with recurrent HCV [22-23]. The specific objectives of this study were to evaluate the efficacy of IL-2RA (Basiliximab) added to the standard immunosuppression regimen in preventing acute cellular rejection (ACR) in HCV-positive liver recipients, and to assess the potential role of IL-2RA in promoting the progression of fibrosis in post-liver transplant recurrent HCV using multiple liver biopsy specimens.

## PATIENTS AND METHODS

Using an electronic pathology database, we reviewed the medical records of all adult patients aged more than 18 years who underwent OLT for end-stage liver disease secondary to chronic HCV infection at the Cleveland Clinic between 1998 and 2006. Patients with at least two post-transplant liver biopsies were included in this study.

Recurrent hepatitis C was defined by the presence of lobular and portal inflammation in patients with detectable HCV RNA, after excluding other causes of histological injury such as graft rejection, biliary disease, vascular complications and confounding viral infections including CMV.

Recipients of living donor grafts were excluded. We also excluded those who have been referred for a second OLT or multi-solid organ transplants during the period from which the study patients were identified.

Demographic data (patient’s age, gender and race), donor age, viral load (serum HCV RNA level) at four-month and one-year post-OLT, viral genotype, and stage of post-transplant liver biopsies, biopsy-proven acute cellular rejection (ACR) and corresponding treatment and CMV viremia that required antiviral therapy were extracted from prospectively collected data for patients with post-OLT recurrent HCV infection seen at the Cleveland Clinic. Additionally, details of antiviral therapy received after liver transplant was obtained from post-OLT HCV treatment-related data sheets filled out prospectively in all liver transplant patients undergoing therapy.

Histological analysis

According to a protocol, liver biopsies were performed on day seven, month four and yearly after OLT. Additional biopsies were performed as clinically indicated. All biopsies were read by dedicated liver pathologists. The Banff schema [19] was used to evaluate ACR, and accordingly defined as “moderate” or “severe” ACR when rejection activity index scores equal or more than five. The stage of hepatic fibrosis was assessed by Ludwig-Batts scoring system [20], and accordingly assigned using a scale of zero to four (F0: absent, F1: portal fibrosis, F2: periportal fibrosis, F3: bridging fibrosis, and F4: cirrhosis). The first biopsy that showed at least stage one fibrosis after OLT was used for the time-to-progression analysis assuming F0 for all patients at the time of OLT. If there was no evidence of fibrosis during post-OLT follow-up, the last biopsy performed was chosen for the time-to-progression analysis. 

Immunosuppression

Induction therapy at Cleveland Clinic consists of two doses of IL-2RA (Basiliximab). The first dose (20 mg) is administered in the intensive care unit, within 12 hours of transplantation. Patients receive the second dose (20 mg) on postoperative day four. All patients transplanted after May 2004 received the IL-2RA induction therapy, whereas those who had OLT before May 2004 had not. All patients received 1000 mg intravenous methylprednisolone in the operating room followed by a tapering dose of intravenous methylprednisolone to 20 mg/day by post-operative day six. This is followed by conventional steroid taper of oral prednisone until discontinued by day 21 following transplantation. 

Calcineurin inhibitors were administered to all patients. MMF was added when side effects precluded full therapeutic dose of calcineurin inhibitors.

Mild ACR was treated by increasing tacrolimus trough level, while corticosteroids were reserved for those with moderate or severe rejection (rejection activity index >5). Patients with biopsy-proven moderate or severe ACR were treated with an intravenous bolus of 1000 mg methylprednisolone followed by tapering dose of intravenous methylprednisolone or oral prednisone over five days. 

Intravenous valganciclovir was administered for a minimum of seven days post-transplantation as prophylaxis against CMV infection. No CMV prophylaxis was given to the sero-negative donor/sero-negative recipient (D^-^/R^-^) combination.

Virological and laboratory assays

Quantitative polymerase chain reaction assay (Roche Cobas Amplicor HCV Monitor Test, v 2.0) was performed to detect HCV RNA four months and one year after OLT. Prospective surveillance cultures were obtained by protocol, and CMV was isolated from buffy coat using conventional cell culture methods. Blood buffy coat was cultured for CMV at weekly intervals during the first two months after OLT and when clinically indicated. In this study, CMV infection was defined as CMV viremia that required treatment with ganciclovir.

Statistical analysis

Descriptive statistics were computed for all variables. These include medians and percentiles for continuous variables and frequencies for categorical variables. Wilcoxon rank sum tests for continuous variables and Pearson’s ϰ^2^ for categorical variables were used to evaluate differences between subjects treated with IL-2RA and those who were not. To assess the association of several baseline variables and liver disease progression, univariate and multivariate Cox proportional hazards analyses were performed. Kaplan-Meier plot was constructed for visual representation of the (unadjusted) association between use of IL-2RA and progression of fibrosis. Recipient’s age, CMV infection, steroid therapy for ACR, antiviral therapy for HCV and IL-2RA induction therapy were included in a multivariate model. A p value <0.05 was considered statistically significant. SAS software ver 9.1 (SAS Institute, Cary, NC) and R software ver 2.4.1 (The R Institute for Statistical Computing) were used to perform statistical analyses.

## RESULTS

Patients' characteristics

A total of 161 patients who had liver transplantation for chronic HCV infection between 1998 and 2006 were identified. Thirty-seven (22%) patients were excluded for having one or more exclusion criteria. The baseline characteristics of our study population are presented in Table 1. The median (25%ile, 75%ile) age of recipients was 55 (50, 59) years with predominant male gender (n=106, 85.5%) and Caucasian ethnicity (n=94, 75.8%). The majority of the patients (80.2%) were infected with HCV genotype 1. 

**Table 1:** Clinical features of the study patients with or without progression of fibrosis after liver transplantation. Continuous variables are presented as median (25%ile, 75%ile).

Factor	ALL (n=124)	IL-2RA (n=70)	Non-IL-2RA (n=54)	P value
Donor age	46.0 (34.0, 62.0)	47.0 (34.0, 62.0)	44.5 (30.5, 47.0)	0.38
Recipient age	55.0 (50.0, 59.0)	53.0 (50.0, 58.0)	56.5 (51.0, 62.0)	0.053
Male	106 (85.5%)	57 (81%)	49 (91%)	0.14
Caucasian	94 (75.8%)	50 (71%)	44 (82%)	0.2
Genotype 1	77/96 (80.2%)	49 (80%)	28 (80%)	0.97
HCV RNA at 4 month (×10^6^)	2.0 (1.0, 4.0)	2.9 (1.0, 5.0)	1.4 (1.0, 2.3)	0.025
HCV RNA at 1 yr (×10^6^)	2.0 (1.1, 3.4)	2.2 (1.2, 3.9)	2.0 (1.0, 2.8)	0.37
HCV therapy	29 (23.4%)	13 (19%)	16 (30%)	0.15
CMV infection	24 (19.4%)	20 (29%)	4 (7%)	0.003
ACR*	53 (42.7%)	20 (29%)	33 (61%)	<0.001
Steroids for ACR	41 (34.1%)	16 (23%)	25 (50%)	0.002
Cyclosporine	16 (13%)	6 (9%)	10 (20%)	0.067
Tacrolimus	96 (81%)	59 (86%)	37 (76%)	0.17
Mycophenolate Mofetil	41 (34.8%)	33 (48%)	8 (16%)	<0.001

* ACR=Acute cellular rejection

All patients had received deceased donor liver allograft with a median (25%ile, 75%ile) donor age of 46 (34, 62) years. The median (25%ile, 75%ile) serum HCV RNA load was 2.0 (1.0 , 4.0) ×10^6^ IU/mL four months after OLT and 2.0 (1.1 , 3.4) ×10^6^ IU/mL one year after OLT. 

Antiviral therapy for HCV (*i.e.*, interferon or pegylated interferon plus ribavirin) was given to 29 (23.4%) patients during the follow-up period. Sustained virologic response (SVR) was achieved in eight of 29 patients. Immunosuppressive therapy was documented for a total of 118 patients and was primarily tacrolimus in 96 (81%) patients, while 16 (13%) received cyclosporine-based regimen. The two treatment groups—IL-2RA and Non-IL-2RA—were comparable with regard to the choice of calcineurin inhibitor. MMF was used in 41 (35%) patients. Biopsy-proven ACR occurred in 53 (42.7%) patients, 41 (34.1%) of whom required steroid therapy. Twenty-four (19.4%) patients developed symptomatic CMV viremia after OLT requiring ganciclovir therapy. 

Hepatitis C viremia

Compared to the patients who did not receive IL-2RA induction therapy, viral load four months after OLT was significantly higher in those who had the induction therapy (median [25%ile, 75%ile] of 1.4 [1.0, 2.3] ×10^6^ *vs.* 2.9 [1.0, 5.0] ×10^6^ IU/mL; p=0.025]. On the other hand, there was no significant difference in HCV RNA at one year post-OLT between the two treatment groups (2.0 [1.0, 2.8] ×10^6^ *vs.* 2.2 [1.2, 3.9] ×10^6^ IU/mL; p=0.37]. 

Acute cellular rejection

The rate of ACR was significantly lower in patients who received IL-2RA than those who did not (20 of 70 [29%] *vs.* 33 of 54 [61%]; p<0.001]. Moderate to severe rejection (defined as rejection activity index > 5) rate was 22.9% in IL-2RA-treated group compared to 50% among those who did not have IL-2RA therapy (p=0.002). 

Predictors of fibrosis progression

In our study population, of 124 patients with recurrent HCV after OLT, a total of 593 post-OLT liver biopsies were performed. The median (25%ile, 75%ile) follow-up time after OLT was 12.1 (6.1, 23.9) months. Overall, the median (25%ile, 75%ile) rate of progression of fibrosis was 0.08 (0.0, 0.17) fibrosis stage per month. Of 124 patients, 76 (61%) had evidence of progression of fibrosis (any type of fibrosis during the follow-up) and 48 (39%) had no fibrosis in liver biopsy. 

**Figure 1:**
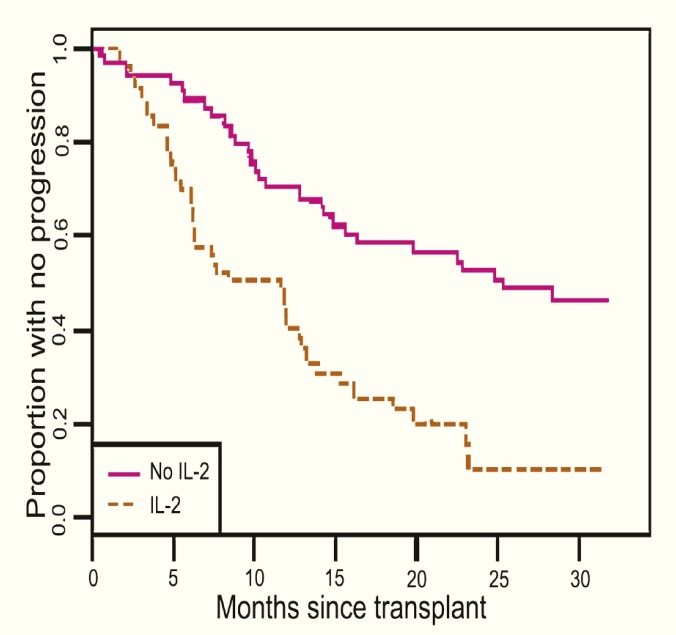
The effect of IL-2 receptor antagonist on the progression of fibrosis in patients with recurrent hepatitis C following liver transplantation

Univariate and multivariate analyses of variables associated with progression of fibrosis in this population are shown in Tables 2A and 2B. In univariate analysis (Table 2A), younger recipient’s age (p=0.02), higher HCV viral load four months after OLT (p=0.002), CMV infection (p<0.001), IL-2RA treatment (p<0.001), HCV antiviral therapy (p=0.017) and steroid therapy for ACR (p=0.043) were associated with higher hazards for the progression of fibrosis after OLT. Kaplan-Meier plot outlining the association between IL-2RA induction therapy and progression of fibrosis is illustrated in Figure 1. 

**Table 2A:** Factors associated with the progression of fibrosis by univariate analysis

Factor	Hazard Ratio	(95% CI)	p value
Donor Age	1.04	(0.96, 1.1)	0.34
Recipient Age	0.83	(0.71, 0.97)	0.02
Gender	1.1	(0.57, 2.2)	0.76
Non-Caucasian ethnicity	1.2	(0.71, 2.0)	0.52
Genotype 1	1.7	(0.82, 3.6)	0.15
HCV RNA at 4 month	1.1	(1.04, 1.2)	0.002
HCV RNA at 1 year	0.97	(0.84, 1.1)	0.71
HCV antiviral therapy	1.8	(1.1, 3.0)	0.017
CMV infection	2.5	(1.5, 4.3)	<0.001
Steroid therapy for ACR*	1.6	(1.02, 2.6)	0.043
Cyclosporine	1.05	(0.54, 2.05)	0.89
Tacrolimus	1.3	(0.70, 2.4)	0.4
Mycophenolate Mofetil	1.08	(0.65, 1.8)	0.77
IL-2RA	2.8	(1.7, 4.6)	<0.001

* ACR=acute cellular rejection.

**Table 2B:** Factors associated with the progression of fibrosis—multivariate analysis

Factor	Hazard Ratio	95% CI	p value
Recipient age (5 yrs increase)	0.86	(0.73, 1.01)	0.069
IL-2RA	3.1	(1.8, 5.3)	<0.001
Steroid therapy for ACR*	2.9	(1.5, 5.8)	0.002
CMV infection	1.6	(0.88, 2.8)	0.12
HCV antiviral therapy	2.8	(1.6, 4.8)	<0.001

* ACR=Acute cellular rejection

After adjusting for recipient’s age, CMV infection, HCV antiviral therapy and steroid therapy for ACR, we found that IL-2RA treatment was associated with a three-fold increase in the hazard of progression of fibrosis (Table 2B). In addition, use of corticosteroids to treat acute rejection (hazard ratio=2.9, p=0.002) remained significantly associated with progression of fibrosis. Patients who received antiviral therapy were more likely to develop post-transplant fibrosis than those who were not treated (hazard ratio=2.8, 95% CI: 1.6–4.8; p<0.001). 

## DISCUSSION

Recurrence of hepatitis C following OLT is a universal complication. It is estimated that 20% to 40% of patients with HCV develop cirrhosis by five years following transplantation secondary to viral infection [1–3]. Among the many factors that may potentially predispose the patient to more severe recurrence of HCV after OLT, immunosuppression is of particular interest. Corticosteroid treatment and lymphocyte depleting agents (*e.g.*, OKT3 and ATG) in patients with recurrent HCV after OLT are associated with a large increase in HCV RNA levels and progression of fibrosis [9–12]. On the other hand, the impact of other immunosuppressive agents including MMF, calcineurin inhibitors, sirolimus and IL-2RA on viral kinetics and progression of hepatic fibrosis after liver transplantation has not been well identified [12–17].

Basiliximab is a humanized monoclonal antibody that blocks the alpha chain (CD25) of IL-2 receptors on activated Th-1 lymphocytes. The CD25 of IL-2 receptor on Th-1 lymphocytes plays a major role in the development of ACR in the transplant patients. The two 20-mg doses of basiliximab (within 12 hours of transplantation and on post-OLT day four) are effective in achieving significant decline in CD25 saturation for over 30 days after OLT—the period when patients are strongly immunogenic. Therefore, basiliximab has been proven effective in decreasing the incidence of ACR in renal and liver transplant recipients [21, 22]. Our study demonstrated that the rate of ACR was significantly (p<0.001) lower in patients with HCV who received IL-2RA induction therapy (basiliximab) compared to those who did not (28.6% *vs*. 61.1%).

The independent impact of IL-2RA on post-transplant HCV infection is still a source of enduring debate. Some of this controversy has been spurred by the fact that IL-2RA decreases the rejection rate and thus the exposure to corticosteroids, both of which are significant risk factors for rapid development and severity of histological recurrence of hepatitis C [9–12]. On the other hand, *in vitro* reports showed that activation of IL-2 receptors on Th-1 lymphocytes plays a significant role in the elimination of HCV-infected hepatocytes. Therefore, it is hypothesized that IL-2 receptor antagonism may favor viral replication and accelerate graft damage. Nelson, *et al* [23] showed that the use of IL-2RA in the early peri-transplantation period may be associated with early recurrence of hepatitis C and more rapid histological progression of the disease. On the other hand, Klintmalm *et al* [24] reported that the use of IL-2RA (daclizumab) has neutral impact on hepatic fibrosis progression at one year following OLT in HCV-positive liver recipients. However, that study investigated the use of daclizumab in a steroid-free regimen compared to a steroid based one. In our study, steroids were administrated to all patients in the IL-2RA and non-IL-2RA treatment groups. Adjusting for recipient age, CMV infection, HCV antiviral therapy and steroid therapy for rejection, we showed that basiliximab induction therapy was independently associated with a three-fold increase in the hazard of progression of fibrosis.

A strong correlation between rejection episodes that were treated with bolus corticosteroids and increased severity of histological recurrence of HCV was well documented in our study. Corticosteroids are known to increase the level of hepatitis C viremia and to be associated with more severe patterns of histologic injury [9–12]. The choice of calcineurin inhibitor (tacrolimus *vs*. cyclosporine) and the use of MMF were not associated with histological progression of HCV after OLT. 

Other factors that potentially cause the progression of fibrosis included high HCV RNA at four months after transplant, and CMV infection, both of which have been identified in several previous studies [6–8]. The effect of donor age did not reach statistical significance, likely because of the young age of most donors enrolled in our study. Unexpectedly, antiviral therapy after OLT was associated with more progression of fibrosis (p=0.001, multivariate analysis). However, it is difficult to assess the role of antiviral treatment after OLT in the progression of fibrosis in our study group. Indeed, patients who were non eligible for antiviral therapy may represent a population with persistently low fibrosis scores regardless of antiviral treatment. Only a randomized trial with a long follow-up could clearly establish the benefit of antiviral treatment on fibrosis.

A major limitation of our study design was that it was based on a retrospective cohort. Outcomes in the study group were compared with a historical cohort that had been transplanted between 1998 and 2004. Future randomized prospective trials are needed to investigate the role of IL-2RA in post-transplant HCV infection. 

Based on these data, the inclusion of basiliximab induction therapy to a calcineurin inhibitor based immunosuppressive regimen in OLT patients with recurrent hepatitis C seems to offer an advantage in terms of lowering the rate of ACR, however it is associated with a higher rate of HCV viremia and more severe histological recurrence of hepatitis C. 
